# Molecular Regulatory Pathways Link Sepsis With Metabolic Syndrome: Non-coding RNA Elements Underlying the Sepsis/Metabolic Cross-Talk

**DOI:** 10.3389/fnmol.2018.00189

**Published:** 2018-06-05

**Authors:** Chanan Meydan, Uriya Bekenstein, Hermona Soreq

**Affiliations:** ^1^Department of Internal Medicine, Mayanei Hayeshua Medical Center, Bnei Brak, Israel; ^2^The Department of Biological Chemistry, The Edmond and Lilly Safra Center for Brain Sciences, The Alexander Silberman Institute of Life Sciences, The Hebrew University of Jerusalem, Jerusalem, Israel

**Keywords:** metabolic syndrome, miRs, ncRNAs, sepsis, SNPs

## Abstract

Sepsis and metabolic syndrome (MetS) are both inflammation-related entities with high impact for human health and the consequences of concussions. Both represent imbalanced parasympathetic/cholinergic response to insulting triggers and variably uncontrolled inflammation that indicates shared upstream regulators, including short microRNAs (miRs) and long non-coding RNAs (lncRNAs). These may cross talk across multiple systems, leading to complex molecular and clinical outcomes. Notably, biomedical and RNA-sequencing based analyses both highlight new links between the acquired and inherited pathogenic, cardiac and inflammatory traits of sepsis/MetS. Those include the HOTAIR and MIAT lncRNAs and their targets, such as miR-122, −150, −155, −182, −197, −375, −608 and HLA-DRA. Implicating non-coding RNA regulators in sepsis and MetS may delineate novel high-value biomarkers and targets for intervention.

## Significance Statement

Sepsis and metabolic syndrome (MetS) both carry a high toll on human health and modern healthcare. Sepsis is the reaction to overwhelming infection in the organism, usually addressed with systemic antimicrobial therapy and systemic supporting measures, whereas MetS is an amalgam of interlinked metabolic disturbances such as obesity, diabetes, hypertension and associated cardiac comorbidities. These seemingly unrelated entities may be regulated by nervous system-mediated interlinked molecular pathways, which can explain the accompanying mal-reaction to pathogens and metabolic challenges. Novel molecular/genomic assessment tools can offer potential new intervention points to consider in future approaches, foreseeing new management modalities.

## Sepsis and MetS Present Escalating Inter-Related Challenges

Sepsis and MetS represent an inter-related escalating disease burden for modern healthcare systems. In spite of increasingly structured approaches, sepsis remains a leading cause of death in hospitals; and MetS is evolving to become a global epidemic, characterized as a combination of obesity, dyslipidemia, hypertension, insulin resistance, and increased thrombosis (Grundy et al., 2004; de Simone et al., 2007). MetS increases the risk for adverse cardiac outcomes such as coronary artery disease, heart failure, cardiac fibrosis and intracardiac malconduction leading to dysfunction and arrhythmias (affected by sympathetic imbalance, changed tissue impedance and changed cardiac electrophysiologic properties; Wilson et al., 2005; Ingelsson et al., 2006; Grassi et al., 2007; de Simone et al., 2007; Suzuki et al., 2008; Wang et al., 2010; Sardu et al., 2014a, 2017). Additionally, MetS elevates the risk for complications of sepsis, possibly relating in part to these maladaptive cardiometabolic alterations (Angus and van der Poll, 2013; Abbasi, 2017). One such dreaded complication of sepsis is intracardiac malconduction and altered electrical properties, exposing patients to life-threatening arrhythmias (Shahreyar et al., 2018). These complications are increasingly being recognized as part of MetS, and may lead to specific device therapies in the future, with discrete molecular pathology-and-response profiles (Marfella et al., 2013; Sardu et al., 2017). Inflammation is key to both sepsis and MetS, with established descriptions of inflammation in the former, and more recent integration into contemporary clinical definitions of the latter (Grundy et al., 2004). Therefore, novel scientific tools are being sought for evolving insights into the underlying biological mechanisms, with impressive attempts at new therapies.

Both sepsis and MetS show increasing interactions with the complex networks of long non-coding RNAs (lncRNAs), microRNAs (miRs) and their targets, compatible with the escalating importance of non-coding RNAs in various pathologies in the neurodegenerative and cardiometabolic arenas (Marfella et al., 2013; Meydan et al., 2016; Salta et al., 2016). Also, the apparent roles for lncRNAs and miRs in immune regulation (Morchikh et al., 2017) highlight their inflammatory association and may lead to the development of novel molecular tools co-regulating these discrete medical entities. Identification of signaling pathways involving environmental and nutritional influences may also pave the way for new interventions as well as shed light on existing therapeutic strategies (Wellen and Thompson, 2012). Sepsis involves an overwhelming systemic inflammatory reaction to infection and represents a complex joint action of pro-inflammatory and anti-inflammatory agents, driven by combined pathogen-specific and host-specific features and the interactions between them (Rittirsch et al., 2008; van der Poll and Opal, 2008). It is characterized by central nervous system (CNS), hemodynamic, cardiac and renal malfunction; adrenergic, glycemic, and coagulation deregulation; and cholinergic/inflammatory imbalance (Angus and van der Poll, 2013), which are largely amplified with underlying comorbidities, and impose high mortality even in modern healthcare settings. A recent call-to-arms by the Global Sepsis Alliance demonstrates this ongoing challenge (Reinhart et al., 2017), and the underlying mechanisms of sepsis have increasingly drawn attention over the last few decades.

Viewing sepsis and MetS as a reflection of acutely imbalanced neuroimmune reactions has spurred an investigation into the specific mechanisms acting jointly with immune machinery and their application in other contexts, such as brain function, which may decline during acute sepsis or recovery (Hanisch et al., 2008; Jackson et al., 2014). This decline involves cortisol decreases in the circulation, due to inhibition of the cholinergic hypothalamic-pituitary-adrenal axis by anxiety and/or changes to associated molecular machinery (Lebow et al., 2012; Galatzer-Levy et al., 2017). Dysfunction of this axis is a key characteristic of both sepsis and MetS (see Supplementary Material). Genomic polymorphisms in cholinergic elements predispose to other mental pathologies, such as drug addictions; therefore, brain malfunction during sepsis is likely to reflect this genomic pathway as well. That cholinergic imbalance is paramount to inflammatory reactions strengthens this assumption (Li and Burmeister, 2009), while raising the issue of personalized cholinergic reinforcement for intervention (Maskos et al., 2005).

In comparison to sepsis, MetS shows a sharply rising prevalence, with a steadily widening presence in many countries, and a risk for coronary and cerebrovascular diseases (Mottillo et al., 2010; O’Neill and O’Driscoll, 2015). Longstanding evidence defines MetS as an inflammatory entity which combines obesity, dyslipidemia, insulin resistance, and hypertension (Ridker et al., 2005), albeit with incompletely understood mechanisms. MetS further complicates the treatment of sepsis, including obesity-related restrictive lung disease and supply-related myocardial ischemia (Type II myocardial infarction), with putative inter-related mechanisms. In this aspect, MetS may present a differently-paced decline of immune-related functions, which parallel those that fail in septic events. This calls for exploring inter-related regulatory pathways controlling these two entities, including non-coding RNA controllers of inflammation that operate well under healthy conditions but may fail in sepsis and MetS. Combining biomedical and RNA-sequencing based evidence indicates that sepsis and MetS (although differing in their clinical nature) share malfunctioning biological domains. This implicates shared miRs and lncRNAs as key cellular regulators of immune-related and other conditions, and as plausible effectors of some of the common clinical and molecular features of sepsis and MetS.

Several miRs are evident in the pathologies of both MetS and sepsis; MiR-122 regulates lipid synthesis and oxidation, and also serves as a biomarker in hepatic ischemia, viral hepatitis and sepsis; MiR-150 is downregulated in sepsis and correlates with its severity as assessed by clinical scoring, but is upregulated in dysglycemia and diabetes mellitus; MiR-182 regulates glucose levels and its metabolism, and is also upregulated in sepsis; and miR-197, −375, −155 and −132 show dual roles in both infection control and MetS (Supplementary Material). An important advantage of miRs is their small size and “druggable” nature, i.e., antisense-oriented oligonucleotides for functional suppression, and vector-based approaches generating “sponge molecules” which act as decoys or miR-mimics for potentiating their activities (Li and Rana, 2014). These strategies may serve as impetus for further elucidation of the roles and possible applications of miRs as direct targets for therapeutics or for exerting regulatory effects on other targets. Several miR-based therapeutics are already in development in the cardio-metabolic context, including an antisense inhibitor of miR-208a, which offers improved recovery from heart failure in animal models (Miragen Therapeutics Inc., Boulder, CO, USA; Montgomery et al., 2011); and preclinical trials for a miR-195 inhibitor, also for heart failure (Servier, miRagen; Li and Rana, 2014).

Recent reports demonstrate a tilted axis of inflammatory mediators and cholinergic machinery which accompany the systemic derangements both in sepsis and MetS. Here, we explore the context of possibly shared elements driving these seemingly distinct clinical spheres. Such discussion becomes increasingly relevant as new molecular and intracellular mechanisms are rapidly being discovered, characterized and harnessed for clinical applications. In turn, new understanding of lncRNAs and miRs, delineation of their interactions, and uncovering their functions in various clinical scenarios can contribute to both the basic and translational research levels of these two conditions. We hope that the current study would spur new initiatives in these contexts, which continue to frustrate modern healthcare systems.

## The lncRNA-miR Link with Inflammation in Sepsis and MetS

MiRs are non-coding regulatory RNA molecules, 100-fold smaller than coding RNAs. Several thousand miRs were identified, many of which are primate-specific (Barbash et al., 2014). LncRNAs as well have been subjected to active evolutionary pressure, adapting them to their diverse functional capacity towards multiple molecular targets and multi-leveled systemic mechanisms (Berezikov, 2011; Barbash et al., 2014). MiRs primarily attach to complementary motifs in target transcripts, thus halting translation of coding RNAs sharing such motifs and exacerbating their degradation. Specific miRs may control shared pathological processes; for example, 11 different miRs were reported to associate with both anxiety-spectrum clinical entities and with MetS, with transcripts of the cholinergic signaling pathway predictably targeted by all of those via the inflammatory triggers shared by these syndromes (Meydan et al., 2016).

**Figure 1:**
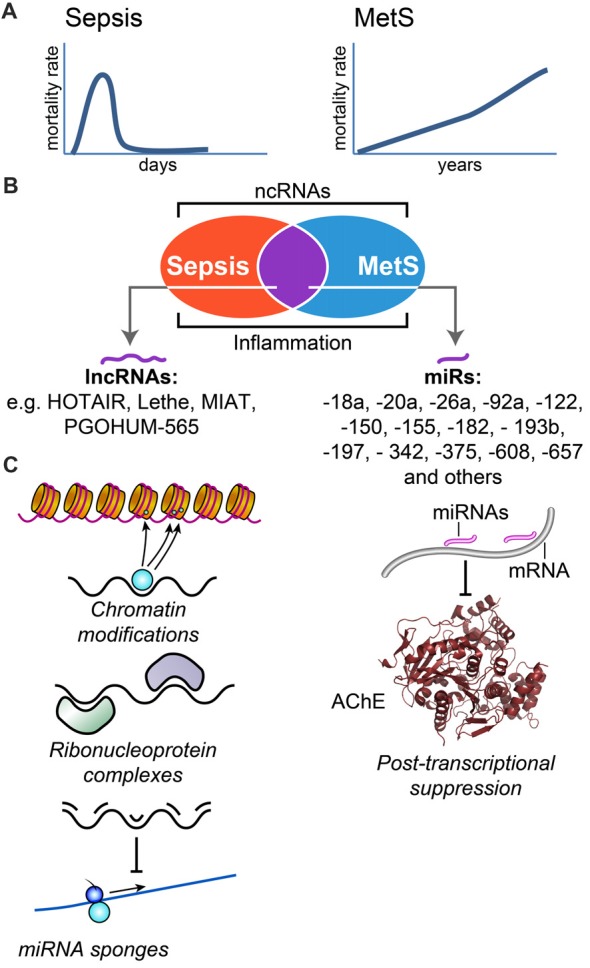
Sepsis and metabolic syndrome (MetS) share ncRNA controllers and inflammation characteristics. **(A)** Scheme of the impact over mortality rate for both sepsis and MetS. Sepsis involves rapid dynamics with mortality rate peaking in the scale of days, whereas entailed mortality from MetS related complications develops over many years. **(B)** Sepsis and MetS are both subject to ncRNA regulation, and share common microRNA (miR) and ncRNA regulators. **(C)** Long non-coding RNAs (LncRNAs; left pane, denoted as black line) exert diverse functions including chromatin modification, ribonucleoprotein complex formation or “sponge” activities, whereas miRs (right hand side, pink lines) primarily suppress their targets via post-transcriptional interaction with short sequence motifs on their target mRNAs, and lead to physiological changes (e.g., cholinergic changes, as a result of acetylcholinesterase (AChE) reduction).

Accelerated accumulation of publicly available RNA-sequencing datasets enables searching for inflammation-related miRs and lncRNAs and the changes occurring in them for sepsis and MetS separately as well as for their shared features (e.g., coagulation, adrenergic/cholinergic imbalance and diabetes, Supplementary Figures S1A,B, Supplementary Table S2). For example, the primate-specific miR-608 controls the MetS-related acetylcholinesterase (AChE; Hanin et al., 2014). A single nucleotide polymorphism (SNP) in the miR-608 sequence limits the risk of post-trauma sepsis (Zhang et al., 2015), indicating that this miR may be related to the aftermath of concussion (Bailes et al., 2013; Zaghloul et al., 2017) and possibly reflecting an apparent link between potential ncRNA controllers of sepsis and MetS at large. Since cholinergic signaling modulates inflammation (Tracey, 2010), ncRNA controllers of parasympathetic functioning are likely candidates for linking between these distinct inflammation-related pathologies and for playing a primary role in the clinical outcome of both sepsis and MetS. Figure 1 schematically presents these predicted inter-related ties between sepsis and MetS, their potential association with lncRNAs and miRs and their predicted mechanisms of action, which are far more diverse for lncRNAs as compared to miRs.

In addition to their individual functions, lncRNAs cooperate with other ncRNAs, such as miRs, both mechanistically (as lncRNA “sponges” suppressing the reacting miRs) and as shared effectors, such as in vascular dysfunction (Yan et al., 2015). So far, 80 miRs were implicated in metabolic disorders (Zampetaki and Mayr, 2012; Arner and Kulyté, 2015). Those are involved in various adipose, lipid, glycemic and vascular impairments, including cardiometabolic clinical ramifications such as heart failure and atrial fibrillation (Marfella et al., 2013; Santulli et al., 2014; Sardu et al., 2015; Xu et al., 2016). In these contexts, miR regulators of organic function may respond to mechanical, electrical and biochemical stimuli, created by changing systemic environments. A notable example of these processes lies in cardiac remodeling, which has classically been viewed as a result of biophysical factors and insults; accumulating evidence implicates miRs as mediators and molecular effectors of this pathophysiology (Divakaran and Mann, 2008; Greco et al., 2012; Barwari et al., 2016). Atherosclerosis, cardiac regeneration, cardiac angiogenesis, cardiac electroconductance and lipid metabolism are additional MetS-related areas with miR involvement (Sardu et al., 2014b; Barwari et al., 2016), including their interaction with established mediators such as angiotensin-II (Pan et al., 2013; Sardu et al., 2016b), and their reaction to specific interventions. In this context, the effect of miRs on adaptive remodeling of cardiovascular organs has been studied, i.e., the association of different miRs’ levels with cardiomyocyte function, features and response to heart failure therapy (Sardu et al., 2014b, 2016a,b; Melman et al., 2015). Some of the metabolically-associated ncRNAs have an apparent role in sepsis as well (Table 1). Briefly, we hypothesized that lncRNA-miR interactions may reflect some of the shared symptoms observed clinically and the inflammatory processes driven by these shared mediators (Supplementary Material). Examples include miR-132, with established roles both in MetS and in the cellular response to viral infections (Lagos et al., 2010; Meydan et al., 2016), and miR-197, dysregulated in adipose tissue dysfunction, and in pulmonary and enteroviral infections (Tang et al., 2015). Such miR responses may both contribute to the host reaction towards infections while influencing seemingly distant clinical phenomena (such as MetS). Scientific tools such as genome wide association scale (GWAS) and RNA-sequencing analyses offer a glimpse towards the evolution of miRs and lncRNAs across different species (Ulitsky, 2016) and can identify associations encountered in current clinical practice, such as sepsis and MetS. Thus, both miRs and short-lived lncRNAs may play key roles in the dynamics and etiology of these conditions (Tani et al., 2012), affect the consequences of concussions and function cooperatively to exacerbate them or protect the body from the corresponding symptoms.

**Table 1:** Non-coding RNA molecules associated with both sepsis and metabolic syndrome (MetS).

Non-coding RNA		Mets/Sepsis	Role	Reference
**microRNAs**	miR-122	MetS	Modulation of hepatocyte-associated proteins (ACC2, SCD1, ACLY, AMP-K) and lipid metabolism	Esau et al. (2006), Elmén et al. (2008) and Zampetaki and Mayr (2012)
		Sepsis	Decreased in sepsis vs. healthy controls and non-septic SIRS, correlates with sepsis mortality	Caserta et al. (2016)
			Increased in chronic liver infection with hepatitis C virus and involved in its pathogenesis	Janssen et al. (2013)
			Marker for abnormal coagulation in sepsis	Janssen et al. (2013), Luna et al. (2015) and Correia et al. (2017)
	miR-150	MetS	Upregulated in adipose and hepatic tissues, and in insulin resistance	Karolina et al. (2012)
		Sepsis	Downregulated in sepsis vs. non-septic SIRS and healthy controls, correlates with SOFA score, predictive of mortality	Vasilescu et al. (2009) and Ma et al. (2017)
			Correlates with proinflammatory cytokines (TNFα, IL-10, IL-18)	Vasilescu et al. (2009)
	miR-182	MetS	Implicated in insulin regulation and diabetes-associated muscle atrophy	Poy et al. (2004), Melkman-Zehavi et al. (2011) and Zhang et al. (2016)
		Sepsis	Upregulated in sepsis in GWAS	Vasilescu et al. (2009)
	miR-197	MetS	Upregulated in adipose tissue	Karolina et al. (2012) and Arner and Kulyté (2015)
		Sepsis	Upregulated in lung infections	Tang et al. (2015)
			Decreased in chronic hepatitis B and enterovirus infections	Tang et al. (2015)
	miR-375	MetS	Suppresses insulin secretion	Zampetaki and Mayr (2012)
		Sepsis	Upregulated in hepatitis B virus infections	Li et al. (2010)
	miR-155	MetS	Involved in the pathogenesis of atherosclerosis and diabetes	Nazari-Jahantigh et al. (2012) and Lin et al. (2016)
		Sepsis	Involved in bacterial infections and hepatitis C-associated liver disease	Correia et al. (2017)
	miR-608	MetS	Involved in cholinergic signaling with implication for hypertension	Hanin et al. (2014)
		Sepsis	SNP is a prognostic marker for reduced risk of sepsis after major trauma	Zhang et al. (2015)
			Interaction with IL-6	Hanin et al. (2017)
**Long non-coding RNAS (lncRNAs)**	HOTAIR	MetS	Implicated in adipocyte differentiation	Wu et al. (2016)
		Sepsis	Promotes TNFα production in cardiomyocites in sepsis, through NF-kB pathway	Wu et al. (2016)
	Lethe	MetS	Leads to inflammatory effects in high-glucose environment	Zgheib et al. (2017)
		Sepsis	Regulates NF-kB and induced by IL-1β and TNFα	Rapicavoli et al. (2013)
	NEAT1	MetS	Regulates PPARg2 splicing during adipogenesis	Chen (2016)
			Mediates miR-140-induced adipogenesis	Gernapudi et al. (2016)
		Sepsis	Induced by herpesvirus infections in a STAT3-dependant manner	Wang et al. (2017)
			Upregulated in Hantavirus infections, downregulation *in vitro* causes impaired immune response, involved in innate immune response through RIG-I pathway	Ma et al. (2017)
	DMRT2	MetS	Suppressed in adipose tissue of obese humans (RNA sequencing)	Liu et al. (2014)
		Sepsis	Induced *in vitro* by LPS stimulation	Liu et al. (2014)
	TP53I13	MetS	Suppressed in adipose tissue of obese humans (RNA sequencing)	Liu et al. (2014)
		Sepsis	Induced *in vitro* by LPS stimulation	Liu et al. (2014)
	Cox2	Sepsis	Induced by Toll-like receptor activation	Carpenter et al. (2013)
	PACER	Sepsis	Involved in assembly of NF-kB	Krawczyk and Emerson (2014)
	Lnc-DC	Sepsis	Regulates dendritic cell differentiation	Wang et al. (2014)
	THRIL	Sepsis	Upregulates TNFα	Li et al. (2014)
	TNFAIP3	Sepsis	Regulated by TNFα, coregulator of NF-kB	Vereecke et al. (2009)

*Shown are lncRNAs and miRs reported to affect MetS and sepsis before and after concussions, including their predicted mechanism of action and associated molecular machinery (See Supplementary Material for additional citations)*.

Several lncRNAs play a role both in sepsis and in MetS. The Lethe lncRNA is involvedplay a role both in sepsis and in MetS in NF-kB regulation, and is co-induced with interleukin (IL)-1β and tumor necrosis factor (TNF)α, and downregulated by high-glucose environment, where it exacerbates the inflammatory cellular profile (Zgheib et al., 2017). This may affect the adverse outcomes of sepsis-induced hyperglycemia and the dysglycemia that accompanies the inflammatory driving force in MetS. In a mouse model of leptin deficiency and diet-induced insulin resistance, the E330013P06 lncRNA is both overexpressed in macrophages and correlated with inflammatory activity (Reddy et al., 2014). HOTAIR is another lncRNA which promotes the inflammatory response to pathogens through the NF-kB pathway (Wu et al., 2016), possibly by interacting with 23 different protein-coding transcripts correlated to adipocyte function. RNA-sequencing of 32 tissue types from 122 healthy human subjects revealed excessive levels of several highly expressed HOTAIR-associated transcripts (over 2-fold excess compared to tissue-wide average) in adipose tissue. These include the Kruppel-like factor 4 (KLF4), Notch homolog 3 (NOTCH3), Snail homolog 1 (SNAI1), Neurotrimin (NTM) and Vimentin (VIM). HOTAIR is also involved in multiple pathways, including the neuronal LSD1/CoREST/REST and PRC2 complexes, and regulates genes in the HOXD locus (Rinn et al., 2007; Tsai et al., 2010; Karpe and Pinnick, 2015). Correspondingly, HOTAIR plays an active role in adipocyte and adipose tissue development, with dispersion between central and peripheral tissues.

In search for putative functional relevance of HOTAIR, we investigated human peripheral RNA-sequencing data from the community acquired pneumonia and sepsis outcome diagnostic study, (CAPSOD, GSE accession number 63042), which enrolled 1152 subjects who presented to emergency care and were stratified into systemic inflammatory response syndrome (SIRS) and four septic groups with escalating severity: uncomplicated sepsis without organ dysfunction; severe sepsis with organ dysfunction; sepsis with hemodynamic collapse (shock); and those dying from sepsis within 28 days from admission. RNA sequencing identified 338 differentially-expressed liver genes between sepsis and non-sepsis SIRS, and 1238 differentially-expressed genes in the groups presenting increasing sepsis severity and outcome. We found several HOTAIR-associated genes to be upregulated in these septic patients, reaching 2–3 fold higher levels compared to non-infectious SIRS; stratifying according to sepsis severity highlighted angiopoietin-2, which is involved in atherosclerosis and in diabetic complications (Park et al., 2014), as increasing with the severity of sepsis, compatible with previous reports (Bopp et al., 2008; Aslan et al., 2017). This supports an inter-related impact for HOTAIR and other MetS-regulated genes, including cholinergic transcripts in the septic liver.

According to the Human Protein Atlas, the subset of MetS-modified genes is elevated 2.7-fold in adipose tissue compared to tissue average in healthy humans, and septic patients in the CAPSOD study show significant upregulation of such genes vs. non-infectious SIRS patients (Table 2). To expand the studied tissue scope, we searched for potential association between the levels of global MetS-modified blood cell coding and non-coding transcripts, and those of liver biopsied tissues from patients with increasing severity of sepsis symptoms. We predicted that transcripts differentially expressed in MetS blood cells would also be modified in the septic liver, and found that many of those transcripts which were changed in MetS blood cells were gradually decreased in septic liver samples with increasing symptoms severity (Supplementary Figure S2).

**Table 2:** Molecular elements associated with the long non-coding RNA (lncRNA) HOTAIR in sepsis and MetS.

Protein symbol	Human protein		Sepsis dataset of CAPSOD study, GSE63042				
Protein name	Atlas, v16.1						
	Tissue-wide average expression (TPM)	Adipose tissue expression (TPM)	Sepsis (mortality) vs. non-infectious SIRS, *t*-test	Septic shock vs. non-infectious SIRS, *t*-test	Severe sepsis vs. non-infectious SIRS, *t*-test	Uncomplicated sepsis vs. non-infectious SIRS, *t*-test	All sepsis vs. non-infectious SIRS, *t*-test
**SAV1**	26.0	35.8	0.12	0.24	0.15	0.33	0.10
Salvador homolog 1							
**KLF4**	44.2	101.0	0.09	0.61	0.51	0.15	0.67
Kruppel-like factor 4							
**EZH2**	6.8	1.8	0.89	0.80	0.63	0.29	0.75
Enhancer of zeste homolog 2							
**SUZ12**							
Suppressor of zeste 12 homolog	19.4	13.9	0.40	0.89	0.94	0.74	0.78
**CHUK**							
Conserved helix-loop-helix ubiquitous kinase	12.6	15.0	0.49	0.50	0.25	0.98	0.66
**IKBKB**							
Inhibitor of kappa light polypeptide gene enhancer in B-cells, kinase beta	8.9	7.4	0.56	0.22	0.09	**0.04**	0.17
**NOTCH3**	20.6	55.8	0.15	0.15	**0.03**	0.21	0.16
Notch homolog 3							
**ELAVL1**	36.4	33.2	0.91	0.21	0.23	0.13	0.21
ELAV-like 1 (Hu antigen R)							
**KDM3A**	35.4	13.7	0.26	0.75	0.72	0.55	0.39
Lysine Demethylase 3A							
**SNAI1**	4.0	9.8	*	*	*	*	*
Snail homolog 1							
**TNF**	0.3	0.3	0.81	0.55	0.68	0.22	0.60
Tumor necrosis factor							
**IGF2**							
Insulin-like growth factor 2 (somatomedin A)	147.6	102.0	*	*	*	*	*
**PCDH10**	4.6	0.6	*	*	*	*	*
Protocadherin 10							
**FOXA1**	9.2	0.3	*	*	*	*	*
Forkhead box A1							
**FOXM1**	6.0	2.4	0.58	0.92	0.50	0.48	0.60
Forkhead box M1							
**CTNNB1**							
Catenin (cadherin-associated protein), beta 1, 88 kDa	154.3	156.3	0.44	**0.07**	**0.02**	0.14	**0.047**
**SETD2**	12.2	10.0	0.44	**0.07**	**0.02**	0.14	**0.047**
SET domain containing 2							
**ASTN1**	3.2	0.3	*	*	*	*	*
Astrotactin 1							
**PCDHA1**	0.2	0.0	0.51	0.39	0.15	0.52	0.94
Ptotocadherin alpha-1							
**MUC5AC**							
Mucin 5AC, oligomeric mucus/gel-forming	10.2	0.0	*	*	*	*	*
**NTM**	8.0	18.3	*	*	*	*	*
Neurotrimin							
**PTK2B**	28.8	16.1	0.22	**0.01**	**0.0004**	**0.01**	**0.0032**
Protein tyrosine kinase 2 beta							
**VIM**	955.0	2578.1	0.18	0.07	**0.0005**	**0.01**	**0.01**
Vimentin

*Unavailable data. Shown are the 23 known mediators associated with HOTAIR; their expression in adipose tissue relative to tissue-average, according to RNA sequencing of tissues from healthy human subjects (The Human Protein Atlas 16.1); and T-test values of their differential blood cells and liver expression levels in various sepsis conditions vs. non-septic SIRS (CAPSOD study, see text), in a descending order (mortality > shock > severe > uncomplicated). Significantly changed expression levels in the CAPSOD database are bolded (P value < 0.1).

The previously unnoticed association between MetS and sepsis transcripts is a striking and non-trivial one, both since we compared blood to liver transcripts and because SIRS may by itself lead to downregulation of some transcripts; indeed, certain MetS-modified transcripts (e.g., angiopoietin 2) showed elevated levels in SIRS compared to sepsis. Of note, testing for sepsis severity-related changes in genes that were reported to interact with HOTAIR showed no significant difference. However, concordant with the predicted cholinergic control of inflammation that may contribute to both conditions, we noted changes in several cholinergic genes. Specifically, the neuronal transcription regulator REST, the muscarinic receptor ChRM3, the nicotinic inflammation-blocking nAChR7 (chrna7) and the obesity-related nicotinic receptor nAChR3 receptor all showed a decline with sepsis severity. In contrast, both the epilepsy-related nicotinic receptor chrnb2 and the allosteric modulator of nicotinic reactions Lynx1 were increased with sepsis severity (Fedi et al., 2008). The apparent links between MetS and sepsis-related blood and liver transcripts provide further support for a functional relationship between the biomedical pathways affected in these two syndromes.

## LncRNAs Involvement in Pancreatic Failure, Diabetes and Ischemic Myocardial Injury

In addition to identifying known disease-related genes, focused studies of specific tissue malfunctioning may detect sepsis and/or MetS-related changes in particular lncRNAs that were not yet defined as directly linked with these syndromes. In this context, pancreatic function is notoriously impaired in both sepsis and MetS. Transcript mapping of human pancreatic islets showed specific lncRNAs associated with beta-cell differentiation and maturation; some of those lncRNAs are glucose-regulated, and were therefore implicated in endocrine function, with two of those (KCNQ1OT1 and HI-LNC45) being dysregulated in type 2 diabetic humans compared to non-diabetics (Korostowski et al., 2012; Morán et al., 2012). Also, KCNQ1OT1 expression predicts heart failure following myocardial infarction, whereas the lncRNA SRA is involved in transactivation of the nuclear receptor PPARg, which contributes to adipocyte differentiation and function (Hubé et al., 2011). The CHAST lncRNA is upregulated in cardiomyocytes during hypertrophic heart disease (Viereck et al., 2016). In comparison, the circulating levels of LncRNA-p5549, -p21015 and -p19461 are downregulated in human obesity compared to non-obese individuals, showing negative correlation with clinical obesity parameters (BMI, waist circumference, waist-to-hip ratio); of these molecules, lncRNA-19461 also associates with glycemic laboratory markers, and is increased towards healthy-control values upon weight loss (Sun et al., 2016).

At the level of protein pathways, KEGG analysis revealed an association of lncRNA-p5549, -p21015 and -p19461 with 10 pathways, with the inflammatory pathway of the innate immune Toll-like receptor signaling being the most enriched (Sun et al., 2016). Also, lncRNA-BATE1 associated differentially in brown vs. white adipose tissue, by forming a functional ribonucleoprotein complex with hnRNP U (Alvarez-Dominguez et al., 2015), indicating functional relevance of RNA metabolism at large. Another brown fat-associated lncRNA molecule, Blnc1, is involved in adipogenesis and adipocyte differentiation and function, and interacts with hnRNP U and with the EBF2 transcription factor associated with adipocyte regulation (Zhao and Lin, 2015). Changes in each or all of these lncRNAs may hence associate with sepsis, MetS or both.

Ischemic myocardial injury is one of the most dreaded outcomes of MetS (Wilson et al., 2005). Elevated levels of the LIPCAR lncRNA associate with poor cardiac prognosis; and serum LIPCAR transcripts are altered in patients following myocardial infarction (Kumarswamy et al., 2014). Cardiac hypertrophy, a manifestation of advanced metabolic damage to the heart muscle also associates with elevated levels of the H19 lncRNA. The latter interacts with miR-675 and CaMKII-delta for its cardiac effects, and may form “sponges” for the let-7 miR (Kallen et al., 2013; Liu et al., 2016). Therefore, both pancreatic failure and ischemic cardiac injury may be susceptible for intricate patterns of ncRNA regulation in septic and MetS patients. Supplementary Table S1 lists further lncRNAs that were so far demonstrated to be involved with MetS, their possible mechanisms of action, and associated mediators.

## Inflammatory Links with Sepsis and MetS-Mis-Regulated ncRNAs

Much of the reported associations between sepsis and MetS relate to their inflammatory links. In sepsis, major inflammatory reaction acutely recruits several systems, whereas in MetS, inflammation is chronic and subclinical, without the classic clinical inflammatory manifestation. Nevertheless, MetS has recently been earmarked as inflammatory in its nature, and shows inflammatory laboratory markers and associated activity of inflammatory mediators (such as CRP, TNFα, IFNγ, and various ILs). Apart from lncRNAs, the human genome includes 8000 lincRNAs (long intergenic non-coding RNAs) which may also contribute to immune system regulation (Carpenter et al., 2013; Rapicavoli et al., 2013). RNA-sequencing data shows that LPS modulates approximately 200 lincRNAs in blood and 60 in adipose tissue, and that those are enriched in NF-kB binding motifs as well as in TLR4 signaling targets, some of which (linc-DMRT2, linc-TP53I13) are expressed in adipose tissue. Also, TP53I13, which is suppressed in obese human subjects (Liu et al., 2014), is elevated in sepsis. In MetS, innate immune functions are co-modified with inflammatory elements: the MetS-upregulated lincRNA Cox2 modulates the expression of inflammatory-associated proteins, such as chemokines and interferon-stimulated genes (Carpenter et al., 2013), and the p50-associated lncRNA PACER leads the assembly of NF-kB transcription complexes, which are major promoters of inflammatory cellular processes (Krawczyk and Emerson, 2014).

Both lincRNAs and lncRNAs regulate inflammation on their own merit, and are themselves influenced by propagating inflammation via feedback mechanisms. The multi-directional nature of the lncRNA-inflammation axis is also evident in the interaction of NF-kB with Lethe, which has immunomodulatory roles over NFkB activities (Rapicavoli et al., 2013). Lnc-DC, another lncRNA governs the inflammatory activities of dendritic cells (antigen uptake, cytokine release) through the paramount STAT3 inflammatory activating pathway, whereas THRIL upregulates the inflammatory agent TNFα that by itself regulates TNFAIP3 (Vereecke et al., 2009; Li et al., 2014; Wang et al., 2014). Some lncRNAs participate in metabolic derangement by influencing other molecular elements involved in these pathologies (Table 2). The multi-leveled interactions of numerous lncRNAs, lincRNAs and miRs with sepsis and MetS highlight their importance for the inflammatory aspects of these syndromes.

## MIAT and miR-608 as Pan-Genomic Links of Sepsis with MetS and CNS Pathologies of Trauma and Mental Illness

**Figure 2:**
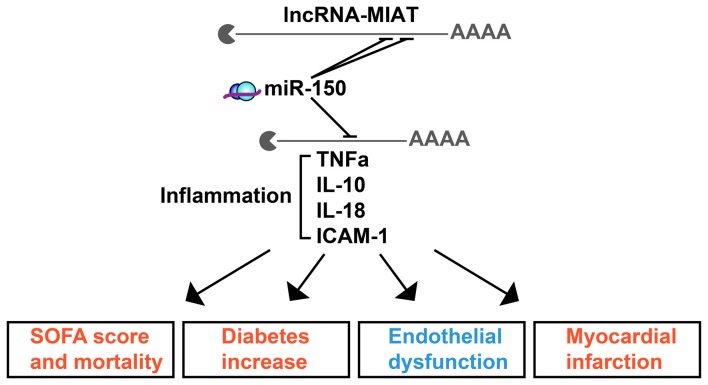
The lncRNA MIAT targets multi-chromosome originated sepsis and MetS-related miRs. MIAT and miR-150 crosstalk exerts inflammatory impact in sepsis and MetS. Shown are selected inflammation-related targets of miR-150 which associate with the clinical characteristics of sepsis (red) or MetS (blue).

The full scope of regulatory ncRNAs likely holds numerous yet-undiscovered connectors between diverse brain and body disease scenarios. One such example is the lncRNA MIAT, mis-regulation of which associates with endothelial dysfunction in diabetes. MIAT derives from chromosome 22 and carries recognition elements for 122 different miRs across numerous chromosomes. Of those, 41, 23 and 13 miRs associate with MetS, sepsis or both, respectively, creating lncRNA-miR-targets networks which may affect a number of related phenotypes (Figure 2). To assess the chromosomal origins and the relative impact of MIAT’s contributions to inflammation, we segregated the predicted MIAT miR targets according to their chromosomal origins and reported clinical associations (Figure 2), and identified a complex profile of MIAT’s contribution to MetS and sepsis. Segregated by their reported association with sepsis and/or MetS, the chromosomal loci of MIAT and its predicted miR targets spanned 11 different chromosomes. For example, in animal models, MIAT interacts with the chromosome 19-originated miR-150-5p; which is an established marker for sepsis staging and outcome in human patients, presumably via its regulation of several inflammation controlling cytokines including TNFα and ILs IL-10, IL-18 (Yan et al., 2015; Figure 2). MIAT’s interaction with miR-150-5p may explain its association with inflammation, severity score and mortality increase in diabetes as well as with the endothelial dysfunction and myocardial infarcts of septic patients, which likely extend to blood-brain barrier malfunctioning.

Molecular links in sepsis-MetS regulation and etiology predict that specific miRs may share targets (coding and non-coding) that are changed in both of these conditions. For example, a SNP in the miR-608-binding motif of the AChE gene associates with several MetS hallmarks (Hanin et al., 2014), whereas another SNP in the miR-608 gene itself limits the risk of sepsis in head injury patients (Zhang et al., 2015). The asymmetric impact of these two SNPs may hence indicate different consequences of AChE and miR-608 changes on MetS and sepsis. The mechanism of action explaining both associations may operate via cholinergic signaling, which can effectively block the inflammatory process via suppression by the homomeric alpha7 acetylcholine receptor of NF-kB-mediated production of pro-inflammatory cytokines (Tracey, 2002; Pavlov and Tracey, 2012). Several cholinergic-related miRs (“CholinomiRs”), including miR-132, miR-608 and miR-211, are functionally involved in inflammatory regulation (Meydan et al., 2016; Bekenstein et al., 2017). However, whether they impact sepsis, MetS or both has only scarcely been explored. Notably, miR-608 targets several inflammation-related transcripts as well as the histocompatibility antigen HLA-DRA (Barbash et al., 2017), which is a biomarker for immunosuppression in sepsis, predictive of survival, and a surrogate marker for GM-CSF experimental therapy of sepsis (Lekkou et al., 2004; Meisel et al., 2009; Cajander et al., 2013). Its association with miR-608 is hence of particular interest.

**Figure 3:**
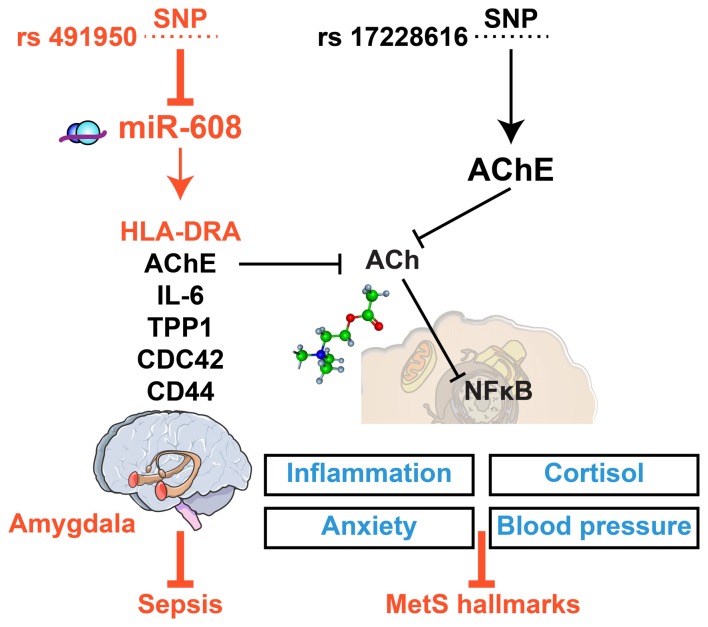
Asymmetric impacts of single nucleotide polymorphisms (SNPs) interrupting miR-608/AChE interaction highlight the relevance for concussions. SNPs in the miR-608 and AChE genes may both interrupt the miR-608/AChE interaction, but the multi-target impact of miR-608 affects the risk of sepsis (red) whereas the AChE SNP relates to MetS (blue). Specifically, the miR-608 SNP (left) may modulate both HLA-DRA and inflammation by interrupting its cholinergic blockade. The AChE SNP (right) may likewise interfere with the cholinergic blockade of NFkB-mediated inflammation, while modulating anxiety, cortisol and blood pressure in MetS patients, which should be highly relevant for the consequences of concussions.

Previous reports revealed molecular cues that might explain the asymmetric impact of the SNP-mediated impairments of the AChE-miR-608 axis: the AChE SNP that interrupts the binding and functioning of miR-608 associates with elevated AChE, inflammation, trait anxiety and blood pressure while reducing cortisol levels in healthy volunteers (Hanin et al., 2014) and inducing hyper-alert reaction to stress in the pre-frontal cortex of minor allele carriers (Lin et al., 2016). The cholinergic imbalance caused by the AChE SNP is hence direct, but its impact is relatively limited. In comparison, the miR-608 SNP may modulate its interaction with both AChE and with numerous other inflammation-related transcripts. These include the amygdala-expressed inflammatory mediators IL-6, CD-44, CDC42 and TPP-1 (Hanin et al., 2014; Lin et al., 2016), which form a pathway resonant with the regulatory ncRNA-cholinergic-inflammatory network. Tilting this balance modulates cortisol levels, with an established role in both MetS and sepsis (Supplementary Material), compatible with targeting the histocompatibility antigen HLA-DRA gene, which is recognized as a hallmark of sepsis (Meisel et al., 2009; Barbash et al., 2017; Figure 3).

To find out the relevance of the imbalanced impact of ncRNAs to blood cells and/or liver transcripts, we sought for predicted targets of miR-608 that associate with MetS and/or sepsis reports, which highlighted its HLA-DRA target as uniquely related to both. Next, we re-searched the CAPSOD dataset for associations between individual transcript changes induced in miR-608 targets with MetS, sepsis or both. Strikingly, we identified parallel changes in the sepsis-related subset of those transcripts derived from patients’ liver tissues compared to healthy controls, and in MetS-derived RNA-sequencing profiles of patients’ nucleated blood cells with their appropriate controls. This is evident in the multi-chromosomal origins of miR-608’s predicted coding and noncoding RNA targets whose levels were modified in these two tissues, and demonstrated considerable similarities in the differential expression patterns of liver and blood cells between MetS and sepsis tissues (Figure 3). In both tissues, those chromosomal domains which showed pronounced response in the septic liver also showed prominent blood cell reactions to MetS, and vice versa, indicating that blood cells RNA-profiles may provide objective clinical assessment of symptoms severity.

Both MetS and sepsis also have mental implications (De Hert et al., 2011; Sonneville et al., 2013) which may relate to another class of lncRNAs, miR-reacting pseudogenes. These lncRNAs have been evolutionarily derived from coding genes, and carry miR recognition elements. SNPs in miR-responding pseudogenes associate with mental diseases such as schizophrenia, autism and bipolar disease, but their putative links to sepsis and MetS have not been explored to date. An example for the interaction between miR-608 and inflammation-regulating lncRNAs lies in the miRs-responding neuronal expressed pseudogene-PGOHUM00000243565 (PGOHUM-565; Barbash et al., 2017). Mechanistically, PGOHUM-565 shares eight different miR recognition elements with HLA-DRA and is predicted to bind the primate-specific miR-608 via its complementary “seed” motif. This may offset the action of miR-608 on numerous coding transcripts, including HLA-DRA and the cholinergic enzyme AChE. Imbalanced interactions of miR-608 with these two targets and others may both tilt the inflammatory response and be mechanistically related to the mental symptoms of sepsis (Cajander et al., 2013), once again highlighting the dissimilarity in the imbalanced ncRNA-target interactions that associate with sepsis and/or MetS.

## Conclusions and Implications for Future Clinical Considerations

We identified genomic and clinical sepsis-related elements that associate with both sepsis and MetS, albeit at different progression rates and with different clinical ramifications. A deeper appreciation of the emerging ncRNAs underlying these shared elements found inflammation-associated molecular agents and cytokines that are affected in both sepsis and MetS. These add up on the established molecules such as Resistin, PAI-1, Adiponectin, Leptin and AGEs (Advanced Glycation End-products) that are prime examples underlying inflammation, putatively accounting for some of the apparent links between sepsis and MetS. The apparent interactions between MetS and the human response to pathogens are becoming evident across the spectrum of these processes. The gut microbiome, which functions mostly as a commensal pathogenic entity, impacts obesity and anti-obesity interventions (Liu et al., 2017). Consistent unfolding of the intricate links between MetS, inflammation and pathogens may further point at novel therapeutic avenues; one recent example is anti-IL-1β antibodies investigated for secondary prevention of myocardial infarction (Ridker et al., 2017). Highly relevant here is the aftermath of repeated concussions, for example in football players (Bailes et al., 2013; Zaghloul et al., 2017).

We suggest and provide evidence for miRs and lncRNAs as potent mediators of inflammatory regulation (including specific examples such as HOTAIR, Lethe, PGOHUM-565) in sepsis and MetS. Specifically, miR regulators of cellular mechanisms including miR-122, −150, −608 and −182 relate to both MetS and sepsis and affect anxiety as well (Supplementary Figures S1C). These lncRNA-miR interactions may form multi-directional regulatory networks, accounting for some of the shared clinical traits and molecular machinery involved. We conclude that altered RNA interactions may modify cellular and brain features in a parallel way to the impact of modified protein networks (Morais et al., 2014), adding a novel regulatory layer that needs to be clinically addressed.

Our current observations highlight upstream regulatory layers to MetS and sepsis progression and outcomes, and even more so, to the associations between them as evident in clinical traits. Based on our current insight, modulating the inflammatory pattern through relevant lncRNA/miR-based networks may be a prime target for sepsis, as an adjunct for antimicrobial and supportive care and for preventing complications after head injury. Hypothesis-driven exploration into the links between MetS and sepsis may hence promote the identification of biomarkers and novel therapies, which are urgently needed.

## Key Concepts

**Non-coding RNAs** are active within genomic domains to alter protein expression and function, and across various clinical spectra, including MetS, sepsis, and head injury risks.

**Cholinergic elements** are neuronally-oriented molecular machinery transcripts with prominent roles in shaping inflammation as a controlled, proportional and adequate process.

The **metabolic syndrome** is an amalgam of chronic pathologic conditions which carry risks for cerebrovascular and other adverse sequelae, including coronary and mental disease.

**Sepsis** is a life-threatening pathological condition in which overwhelming inflammation is ensued in order to avert infections, with several major organic derangements occurring as a result.

## Author Contributions

HS, CM and UB all participated in the information collection, data mining and manuscript design.

## Conflict of Interest Statement

CM receives consultation fees from Raziel Therapeutics, Ltd. The other authors declare that the research was conducted in the absence of any commercial or financial relationships that could be construed as a potential conflict of interest.

## Abbreviations

AChE: acetylcholinesterase
AGEs: advanced glycation end-products
CAPSOD: community acquired pneumonia and sepsis outcome diagnostic study
CholinomiRs: cholinergic-related miRs
CNS: central nervous system
GWAS: genome wide association scale
KLF4: Kruppel-like factor 4
lncRNAs: long non-coding RNAs
MetS: metabolic syndrome
miRs: microRNAs
NOTCH3: notch homolog 3
NTM: neurotrimin
SIRS: systemic inflammatory response syndrome
SNAI1: snail homolog 1
SNP: single nucleotide polymorphism
VIM: vimentin.
