# Supplementary material for: Molecular Regulatory Pathways Link Sepsis With Metabolic Syndrome: Non-coding RNA Elements Underlying the Sepsis/Metabolic Cross-Talk
Source: Front Mol Neurosci. 2018 Jun 5;11:189. doi: 10.3389/fnmol.2018.00189 (PMC5996041; doi:10.3389/fnmol.2018.00189)
Supplement: Supplementary file 1 [file Presentation_1.PDF]

Non-coding RNA co-regulators of inflammation link sepsis with Metabolic Syndrome

Chanan Meydan [1,2*], Uriya Bekenstein[3*], and Hermona Soreq[3, **]

Supplementary biomedical material

Apart from the molecular aspects, the apparent association between sepsis and MetS is reflected in numerous biomedical reports, as is detailed and referenced below; The inflammatory mal-balance in both MetS and sepsis presents clinically with alterations and disruption of homeostasis in multiple systems, and specifically in coagulation abnormalities [1-9], alterations in glucose homeostasis[10-17], and adrenergic dysfunction [18-20]. This is further elaborated below.

Inter-related derangement in sepsis and MetS indicates shared mechanisms of action

Sepsis entails systemic reaction to infection and, by definition, involves distant organs, with an inflammatory surge evident in the clinical and hematology pictures and reflected by molecular biomarkers. MetS is likewise an inflammatory entity, reflecting chronic and ongoing organic insufficiency. This is evident in septic patients by elevated inflammatory markers such as C-reactive protein (CRP), which signals increased cardiovascular risk [21]; and in MetS as elevated levels of established inflammatory cytokines such as IL-6 and TNFa [22]. The role of inflammation intertwined with MetS has been extensively studied in recent years and contemporary view holds MetS as an inherently pro-inflammatory condition [23]. The inflammatory mal-balance in both MetS and sepsis presents clinically with alterations and disruption of homeostasis in multiple systems. While in sepsis this disruption is massive and life-threatening in acute settings, MetS results in chronic disruption that may result in gradual deterioration, such as chronic diabetic renal disease or congestive heart failure. Recently emerging mechanisms form promising links between sepsis and MetS, including elevated levels of the pro-inflammatory agent resistin which correlate

to the severity of disease and prognosis of sepsis in septic shock patients [24] as reflected in serum lactate, creatinine and D-dimer, all surrogate markers of end-organ damage. Resistin is released from monocytes after exposure to septic stimulus (LPS, lipopolysaccharide), and triggers the expression of the ICAM-1 adhesion molecule, presumably facilitating leukocyte transmigration [25]. Also, resistin levels increase in obesity, coronary artery disease and diabetes where they correlate to those of CRP and other inflammatory markers [25-27]. Importantly, these studies highlight shared pathway(s) between sepsis and MetS. Additional system-level pathologies may underlie other common aspects for these clinical situations.

Coagulation

Coagulation is the process of blood clotting which is naturally induced to avert hemorrhage via humoral mediators intrinsic to plasma. Together with platelet aggregation, coagulation sustains a delicate balance between multiple factors creating complementary effects to maintain circulatory volume. Sepsis disturbs the homeostasis between endogenous pro-coagulants and anti-coagulant agents. Protein C and D-dimers, both biomarkers of increased coagulation activity, are imbalanced in approximately 90-100% of septic patients. Factor XII, perkallikrein, and high molecular weight kininogens are all active during sepsis [1]. In approximately half of septic patients, coagulation disturbances culminate clinically in disseminated intravascular coagulation (DIC), which reflects a widespread activation of coagulation agents, fibrin deposition, thrombotic events and concurrent bleeding [1, 2]. The end results are deep venous thrombosis which may cause embolism, micro-angiopathic blockage causing ischemia of perfusion-dependent organs (such as the kidney), and local thrombophlebitis as a nidus for pathogens, coronary events, and cerebrovascular events. Thus, septic patients share common adverse life-threatening sequels.

At the molecular level, clotting abnormalities during sepsis are intimately linked to inflammation. Major inflammatory cytokines such as IL-1, IL-6 and TNFa have pro-coagulation activities [28, 29], whereas pro-coagulant agents such as thrombin induce the activity of adhesion molecules (E-

selectin, P-selectin, ICAM-1, and VCAM-1) and inflammatory-cell chemotaxis (via MCP-1) [30]. Monocytes and macrophages show upregulated tissue factor expression, resulting in activation of the clotting cascade [1]. Fibrin induces IL-8 expression, which is active in leukocyte chemotaxis [3]. Thrombin signaling changes leukocyte morphology [31], shifting the fine balance from anti- to pro-coagulation, which may promote sepsis-related entities such as venous thrombosis and DIC. Additionally, sepsis involves elevated blood platelet levels, and platelets express P-selectins which are important adhesion molecules for leukocytes [4].

While the relation between sepsis and coagulation is clearly established, evidence is only recently emerging regarding the impact of coagulation on MetS. Specifically, obese people show increased risk for venous thrombosis [5]. Pro-coagulant activity, including fibrinogen, factor VII, factor VIII, vWF, and plasminogen activator inhibitor are all modulated in obesity [6]. Adipose tissue, which has been recognized as hormonally active (with alterations in MetS), expresses plasminogen activator inhibitor-1 (PAI-1) in correlation with obesity [7, 32]. Moreover, PAI-1 levels are influenced by insulinergic, steroidal, fatty acid, and inflammatory (TNFa) stimuli [32]. Diabetes status also predisposes for thrombosis, with a shift towards a pro-thrombotic molecular profile and platelet dysfunction [33] and PAI-1 involvement [34]. These changes may explain part of the increased thrombotic risk incurred by MetS patients, manifesting in venous and arterial thrombosis events. Another possible shared mechanism may relate to protein C, a regulator molecule for coagulation; protein C deficiency is associated with worse prognosis in sepsis [8], so much so that recombinant activated protein C was found to reduce sepsis mortality [9]. In obesity, protein C is elevated and its levels decrease with weight loss [35], possibly attributing its accumulation in MetS to a self-protective mechanism.

The role of non-coding RNA molecules is also evident in hemostasis, both in platelet function and in coagulation. Several proteins with established roles in hemostasis have been associated with miRs and with SNPs in either those miRs or their targets; both the MIRANDA and

TARGETSCANS miR-targeting algorithms predict miR-34a, miR-449b and miR-513a-5p to target coagulation factor VIII, and miR-154 to target coagulation factor XI [36]. Non-coding RNAs have also been implicated in platelet dysfunction [37]. Furthermore, other miRs have been found to be dysregulated in MetS-associated thrombosis pathologies such as ischemic cerebrovascular accidents [38]. Conversely, miR-122, which has been associated with MetS, has also been implicated in sepsis and increased coagulation in sepsis [39].

Disglycemia

Diabetes and insulin resistance are integral and well-established components of MetS, and glycemic status disruption in sepsis has long been observed but so far less explained. Hyperglycemia and highly variable glucose levels during sepsis are predictive of increased mortality, but the benefits of glycemic control in sepsis are less well established. Three multi-center trials, NICE-SUGAR [40], GLUCONTROL [41] and VISEP (which was specific for sepsis) [42], found no advantage in tight glycemic control in intensive care units. The results of these trials, along with evidence of association of hypoglycemia with increasing mortality in intensive care units, resulted in high debate regarding glycemic control and management in these clinical situations [10, 11].

At the molecular and cellular levels, glycemic derangement appears to affect the immune system's function, with reduced neutrophil activity including phagocytosis, despite an observed surge in pro-inflammatory cytokines (IL-6 and TNFa) [12, 13] . This surge has been attributed by some to the effect of glycemic hyper-osmolarity, arguing some reversal of the pro-inflammatory-anti-inflammatory state back to balance [13]. Other mechanisms have been implicated, such as the reduction of advanced glycation end products (AGEs) which are metabolites associated with hyperglycemia [14]. While the exact mechanisms of the effect of hyperglycemia, hypoglycemia and insulin on sepsis are unclear, it is evident that all of these elements have an effect on the molecular machinery driving the host reaction during sepsis, and that some of them play an established role in the pathophysiology of MetS. For example, AGEs have associated receptors and

are implicated in diabetes-related vascular dysfunction, structural derangement, and clinical diabetes-associated morbidity [15]. The AGE-associated machinery has been shown to interact with inflammatory molecular components such as NF-kB, TNFa and IL-6 [16]. Cellular inflammatory activity is also influenced by AGEs-mediated modulation of macrophage activity and transmigration [15]. Consequently, the inflammation-controlling molecular machinery responds to alterations in the glycemic status, with possible relation to the inflammation-associated clinical outcomes. AGEs can also shift endothelium from anticoagulant to pro-coagulant properties via reduced thrombo-modulating activity and increased tissue-factor expression [16, 17]. This may also tie the glycemic imbalance with coagulopathy, as both of these conditions are observed in both sepsis and MetS. Other possible links are the reduced levels of adiponectin and leptin in MetS [43]; such reductions also associate with worse prognosis in sepsis [44, 45]. Taken together, these reports indicate that acute glycemic derangement in sepsis may reflect cumulative failure at the physiological, cellular and molecular levels with parallel chronic glycemic damage as that observed in diabetes mellitus, with AGE as a possible molecular link between these phenomena.

Several miRs have been implicated in dysglycemia in MetS [46], and are emerging as important markers in diabetes and its complications. MiR-150, -182, -375, -155 have an established role in diabetic pathogenesis, as depicted in Table I and the main text. In addition, the Lethe lncRNA has also emerged as involved in diabetes mellitus and inflammation-associated effects of hyperglycemic environments; this forerunner example identifies lncRNAs as having a possible contribution to diabetes and diabetes-associated complications.

Adrenergic Dysfunction

Septic patients often display disrupted adrenergic function, including a variety of related mechanisms reflecting systemic recruitment of cortisol via decreased metabolism and clearance by inactivating enzymes (e.g. 11beta-HSD2) [18] . The increase in circulatory cortisol is a hallmark of sepsis, but the pattern associated with its outcomes has been controversial for many years.

Notably, both increased and decreased circulatory cortisol have been associated with adverse outcomes [19]. Several markers of adrenal malfunction have been noted during sepsis, such as increased cortisol levels at various circadian time points, variability along the course of disease, and reaction to hormonal stimulation (corticotropin), together creating a complex, hard-to-interpret picture. This complexity led to controversy in defining appropriate measurements for assessing adrenal function and in their interpretation during sepsis [19, 47, 48]. Treatment of sepsis with exogenous corticosteroids has been practiced by clinical centers, but was extensively studied in randomized control trials only in recent years. These efforts include studies by [49] and the CORTICUS [50] and HYPRESS [51] studies, with variable results. Consequently, adrenal function during sepsis remains a poorly-understood phenomenon with controversial clinical significance and applicability.

MetS as well has been associated with modified circulatory cortisol levels , which depend on altered fat distribution and the presence of other comorbidities, and are ameliorated with weight loss [20, 52]. Specifically, glucocorticoids have an established function in glycemic and lipid metabolism [53], with glucocorticoid-associated molecular machinery showing a differential distribution between visceral and peripheral adipose tissue, and implications on obesity and insulin resistance [54, 55]. This underlines the notion that the hippocampus-pituitary-adrenal (HPA) axis has a prominent role in the pathophysiology of MetS [20]. In addition to the hypercoagulability in MetS, coagulation factors (XII, XI, IX, VIII), plasminogen and alpha2-antiplasmin are all induced by cortisol excess [56]; high circulatory fibrinogen, homocysteine, thrombin, cysteine and PAI-1 most likely form the molecular aspect of the hypercoagulability observed clinically in patients with increased cortisol levels [57].

The HPA axis, and specifically the adrenal glands' function, has also been associated with several miRs, which in humans includes miR-24, which regulates the expression of adrenal CYP11B1/2 proteins [58]. Apart from physiologic function, miR-379 levels correlate with serum cortisol and

lipid levels in obese humans, and inhibition of miR-379 abolishes dyslipidemia in obese mice [59]. The lncRNA SRA, which is implicated in MetS as described above, co-activates glucocorticoid receptors and is involved in steroidogenesis through co-activation with Dax-1 [60].

Non-coding RNAs as potential links between sepsis and MetS

MicroRNA molecules (miRs) have been shown to be associated with various pathological processes, including anxiety disorders, neurodegenerative pathologies, MetS and sepsis. Evidence shows miRs influencing and regulating pathological pathways, shared across seemingly distinct pathological spheres and creating putative associations between them. In this respect, we have recently shown shared associated miR and cholinergic machinery operating in anxiety disorders and MetS [23]. In the present context, several miRs can be identified in implicating both sepsis and MetS, representing shared regulating mechanisms. These are outlined below and in the main text.

MiR-122 regulates lipid metabolism, especially in hepatocytes [61], where it controls the expression of enzymes associated with lipid storage, synthesis and oxidation including acetyl-CoA carboxylase beta (ACC2), stearoyl-CoA desaturase (SCD1), ATP citrate lyase (ACLY), and AMP activated protein kinase (AMPK) [62]. In both mice and primates, inhibition of miR-122 causes a reduction in total plasma cholesterol, LDL, HDL, apolipoprotein AI, and apolipoprotein B – all of which are important factors in the human lipid profile [63]. In addition, miR-122 is a biomarker for liver damage in hepatic ischemic injury [64] and has a key role in the pathogenesis of chronic liver infection with viral hepatitis [65]. Sequestration by antisense agents (Miravirsen, Roche) is a suggested therapy for the latter [65-67]. Septic human patients show declined serum levels of miR-122 relative to healthy controls and patients with the non-septic, non-infectious Systemic Inflammatory Response Syndrome (SIRS) [68]. This evidence, combined with the assumption that miR-122 levels may reflect hepatic injury during sepsis, underlines it as a candidate biomarker and therapeutic target for this condition and its prognosis.

MiR-150 is a critical regulator of inflammatory cell formation, and specifically B-cell maturation [69]. It is downregulated in sepsis relative to SIRS or healthy controls [70] and is hence considered a biomarker for sepsis. Decreased levels of miR-150 also correlate with sepsis severity as assessed by the SOFA score (Sepsis-related Organ Failure Assessment, integrating circulatory, respiratory, hematologic, metabolic parameters) and the risk of mortality [71]. In septic patients, miR-150 levels inversely correlate with the levels of pro-inflammatory cytokines such as TNFa, IL-10 and IL-18 [71]. Also, miR-150 is upregulated in type 2 diabetes mellitus, in adipose tissue and in the liver [72], and the adipose tissue from miR-150 knockout mice shows exacerbated inflammation and increased insulin resistance, with several target genes adamant in this process (Elk1, Etf1, and Myb) [73].

MiR-182, expressed specifically in human pancreatic islets [74], controls insulin and glucose utilization. In mice studies, it operates through the Sox6 and Bhlhe22 pathways [75-77]. In genome-wide miR profiling of septic patients, miR-182 is upregulated compared to healthy controls [71]. Additional miRs with roles in both MetS and sepsis include **miR-197**, which is upregulated in the circulation and adipose tissue of patients with MetS [78, 79], and is also modulated in patients under viral infections [80]; **miR-375,** which suppresses insulin secretion [62], and is also elevated in hepatitis B viral infections [81]; and **miR-155**, which in mice is important for immune defense, specifically against hepatitis C virus [67, 82], while also being involved in the pathogenesis of atherosclerosis [83] and diabetes [84]. The anti-inflammatory **miR-132** which suppresses AChE, among other targets, potentiates the cholinergic blockade of inflammation [85], and is also causally involved in the MetS-linked hepatosteatosis [86].

With more than 10,000 identified transcripts, lncRNA molecules form an intriguing emerging group of non-coding RNAs. The modes of action of the great majority of them are still not understood, but those few mechanisms that are known are far more diverse than those of miRs and span chromatin modifications of histone activity, formation of ribonucleoprotein complexes

controlling regular and alternative splicing, transcription factor activity and mRNA stability [87, 88]. LncRNAs may further operate post-transcriptionally to modulate gene expression [87], and were implicated in embryonic tissue development (such as skeletal and cardiac muscle), malignant and aging processes. Yet more recently, lncRNAs emerged as having roles in sepsis [88] and MetS, e.g. with effects in cardiac remodeling processes [89]. A recent example is that of the lncRNA NEAT1, which forms large multi-component three dimensional structures with locus-oriented, spatial action on innate immunity [88, 90]; this non-coding molecule is involved in MetS through adipogenesis regulation [91, 92] and may also contribute to the immune response to viral infections [93, 94]. Other lncRNAs exert epigenetic modification, transcription regulation [95], and cell-cycle control [96] or operate as decoy receptors to block upregulation of their target transcripts. To perform their roles, these upstream genomic regulators cross-talk with each other and with the smaller miRs, forming agile molecular devices which are rapidly transcribed and degraded, and do not necessarily depend on translation. Additionally, some lncRNAs can directly modulate the activity of miRs [90, 97], and genome-wide association studies (GWAS) identified disease-associated SNPs in lncRNA genes, which affect networks with miRs and their coding targets [98].

Supplementary Figures and Tables

Table S1: Non-coding RNA molecules associated with both sepsis and MetS.

**Non-coding RNA**		**Mets / Sepsis**	**Role**	**Reference**
microRNAs	miR-122	MetS	Modulation of hepatocyte-associated proteins (ACC2, SCD1, ACLY, AMP-K) and lipid metabolism	[61-63]
		Sepsis	Decreased in sepsis vs. healthy controls & non-septic SIRS, correlates with sepsis mortality	[68]
			Increased in chronic liver infection with hepatitis C virus and involved in its pathogenesis	[65]
			Marker for abnormal coagulation in sepsis	[65-67]
	miR-150	MetS	Upregulated in adipose and hepatic tissues, and in insulin resistance	[79]
		Sepsis	Downregulated in sepsis vs. non-septic SIRS and healthy controls, correlates with SOFA score, predictive of mortality	[71, 94]
			Correlates with proinflammatory cytokines (TNFa, IL-10, IL-18)	[71]
	miR-182	MetS	Implicated in insulin regulation and diabetes-associated muscle atrophy	[75-77]
		Sepsis	Upregulated in sepsis in GWAS	[71]
	miR-197	MetS	Upregulated in adipose tissue	[78, 79]
		Sepsis	Upregulated in lung infections	[80]
			Decreased in chronic hepatitis B and enterovirus infections	[80]
	miR-375	MetS	Suppresses insulin secretion	[62]
		Sepsis	Upregulated in hepatitis B virus infections	[81]
	miR-155	MetS	Involved in the pathogenesis of atherosclerosis and diabetes	[83, 84]
		Sepsis	Involved in bacterial infections and hepatitis C-associated liver disease	[67]
	miR-608	MetS	Involved in cholinergic signalling with implication for hypertension	[99]
		Sepsis	SNP is a prognostic marker for sepsis after major trauma	[100]
			Interaction with IL-6	[86]
Long non-coding RNAS (lncRNAs)	HOTAIR	MetS	Implicated in adipocyte differentiation	[101]
		Sepsis	Promotes TNFa production in cardiomyocites in sepsis, through NF-kB pathway	[101]
	Lethe	MetS	Leads to inflammatory effects in high-glucose environment	[102]
		Sepsis	Regulates NF-kB and induced by IL-1b and TNFa	[103]
	NEAT1	MetS	Regulates PPARg2 splicing during adipogenesis	[91]
			Mediates miR-140-induced adipogenesis	[92]
		Sepsis	Induced by herpesvirus infections in a STAT3-dependant manner	[93]

			Upregulated in Hantavirus infections, downregulation in vitro causes impaired immune response, involved in innate immune response through RIG-I pathway	[94]
	DMRT2	MetS	Suppressed in adipose tissue of obese humans (RNA sequencing)	[104]
		Sepsis	Induced in vitro by LPS stimulation	[104]
	TP53I13	MetS	Suppressed in adipose tissue of obese humans (RNA sequencing)	[104]
		Sepsis	Induced in vitro by LPS stimulation	[104]
	Cox2	Sepsis	Induced by Toll-like receptor activation	[105]
	PACER	Sepsis	Involved in assembly of NF-kB	[106]
	Lnc-DC	Sepsis	Regulates dendritic cell differentiation	[107]
	THRIL	Sepsis	Upregulates TNFa	[108]
	TNFAIP3	Sepsis	Regulated by TNFa, coregulator of NF-kB	[109]

Caption for table S1: Shown are lncRNAs and miRs associated in literature with effects relating to both MetS and sepsis, including their predicted mechanism of action and associated molecular machinery.

Table S2: LncRNAs associated with MetS

**LncRNA**	**Metabolic Syndrome**		
	**Effect**	**Mechanism of Action**	**References**
HOTAIR	Implicated in adipocyte differentiation	Interacts with PRC2 and LSD1/CoREST/REST complexes and suppresses gene expression; "scaffold" function that binds together protein complexes to establish a repressive chromatin state; regulates transcriptional silencing of genes in HOXD locus	[110-112]
KCNQ1OT1	Dysregulated in pancreatic islets in diabetes mellitus; Predictive of heart failure following myocardial infarction	Affects chromatin conformation and expression of KCNQ1, involved in neonatal cardiac development (mouse)	[113-115]
HI-LNC45	Dysregulated in pancreatic islets in diabetes mellitus	Poorly elucidated	[114]
P5549	Downregulated in human obesity	Unelucidated implication on Toll-like receptor signaling pathway	[116]

P21015	Downregulated in human obesity	Unelucidated implication on Toll-like receptor signaling pathway	[116]
P19461	Downregulated in human obesity, correlating with glycemic parameters, with improvement upon weight loss	Unelucidated implication on Toll-like receptor signaling pathway	[116]
BATE1	Expressed in adipocyte with different expression profiles between white and brown adipose tissues	Interaction with hnRNP U and may form a ribonucleoprotein complex	[117]
Blnc1	Involved in adipocyte differentiation and adipocyte function	Interaction with the EBF2 adipocyte-associated transcription factor, and hnRNP U which forms a scaffold for this interaction	[118]
LIPCAR	Associated with recovery patterns after myocardial infarction	Poorly elucidated	[119]
H19	Associated with cardiac hypertrophy	Effect mediated by miR-675 and CaMKII-delta; H19 acts as a molecular sponge for let-7 miRs	[104, 120]
ANRIL	Associated with pro-atherogenic cellular functions and heart failure	Trans-regulation through Alu motifs and binding of epigenetic effector proteins	[115]
SRA	Promotes mesenchymal precursor cell differentiation into adipocytes, increases glucose uptake, involved in adipocyte function	Transactivation of PPARg (nuclear receptor involved in glucose metabolism and adipocyte differentiation and function); involvement in cell cycle, insulin and TNFa pathways	[121]
MIAT	Associated with endothelial dysfunction in diabetes mellitus	Act as "sponge molecule" for miR-150-5p and interacts with VEGF in endothelial cells (animal models)	[122]

Caption for table S2: Shown are lncRNAs associated in literature with effects relating to MetS, including their predicted mechanism of action and molecular machinery.

Figure S1

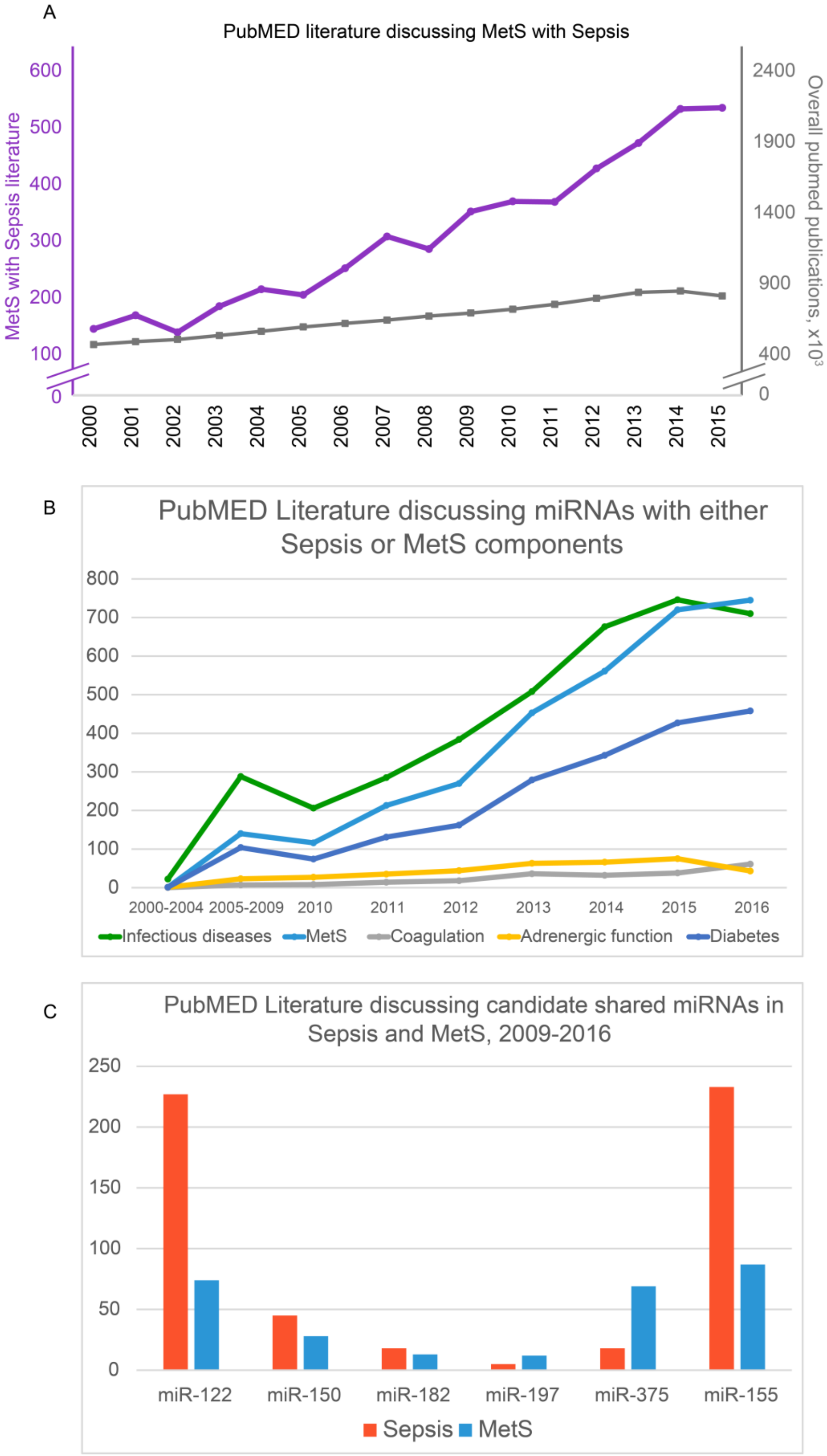
A
PubMED literature discussing MetS with Sepsis
MetS with Sepsis literature
Overall pubmed publications, x10³
600
500
400
300
200
100
0
2400
1900
1400
900
400
0
2000
2001
2002
2003
2004
2005
2006
2007
2008
2009
2010
2011
2012
2013
2014
2015
B
PubMED Literature discussing miRNAs with either Sepsis or MetS components
800
700
600
500
400
300
200
100
0
2000-2004
2005-2009
2010
2011
2012
2013
2014
2015
2016
Infectious diseases
MetS
Coagulation
Adrenergic function
Diabetes
C
PubMED Literature discussing candidate shared miRNAs in Sepsis and MetS, 2009-2016
250
200
150
100
50
0
miR-122
miR-150
miR-182
miR-197
miR-375
miR-155
Sepsis
MetS

Caption for Figure S1: PubMED Literature discussing MetS links to Sepsis (A), either Sepsis or MetS components (B) or shared miRNAs in Sepsis and MetS, 2009-2016 (C).

A. A PubMED online search with the string "((metabolic syndrome) OR diabetes OR dysglycemia OR hyperglycemia OR obesity OR overweight OR hypertension OR dyslipidemia OR hyperlipidemia) AND (sepsis)" was performed on May 13th, 2017.

B. PubMED Literature discussing miRs with either Sepsis or MetS components. A PubMED online search with the strings "(sepsis OR infection) AND (microRNA OR miRNA)" and "sepsis AND (microRNA OR miRNA)" for infectious diseases, "((metabolic syndrome) OR diabetes OR dysglycemia OR hyperglycemia OR obesity OR overweight OR hypertension OR dyslipidemia OR hyperlipidemia) AND (microRNA or miRNA)" for MetS, "(coagulation OR hemostasis OR haemostasis) AND (microRNA or miRNA)" for coagulation disorders, "(adrenal OR addison OR corticosteroid) AND (microRNA OR miRNA)" for adrenergic disorders, and "(diabetes OR dysglycemia OR hyperglycemia) AND (microRNA or miRNA)" for diabetes, dated May 13th, 2017.

C. PubMED Literature discussing candidate shared miRNAs in Sepsis and MetS, 2009-2016. A PubMED online search for miR-122, -150, -155, -182, -197, -375, in association with MetS and sepsis: "(miR-X OR miRNA-X OR microRNA-X) AND ((metabolic syndrome) OR diabetes OR dysglycemia OR hyperglycemia OR obesity OR overweight OR hypertension OR dyslipidemia OR hyperlipidemia)" and "(miR-X OR miRNA-X OR microRNA-X) AND (sepsis OR infection)", respectively. "X" denotes the miR number as elaborated.

Figure S2

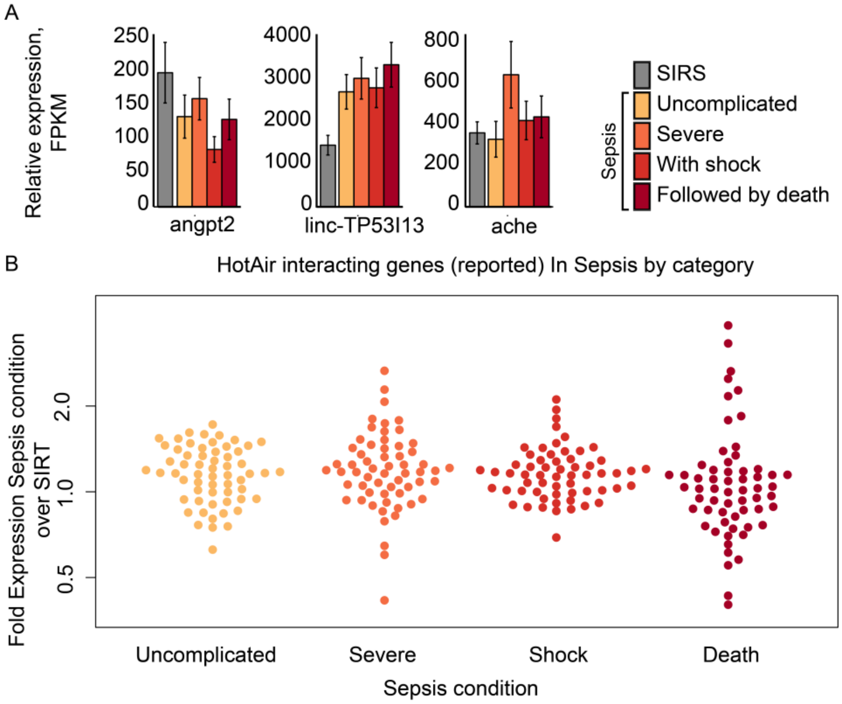

Caption for figure S2: Bidirectional sepsis-related changes in the CAPSOD dataset.

A Individual examples of changes in angiopoetin2, linc-TP53I13 and AChE in the noted disease stages.

B Distributions of changes in the levels of blood cells' transcripts from SIRS patients compared to the changes in liver transcript levels from sepsis patients with increasing symptoms severity (CAPSOD dataset; see main text for details).

Supplementary Bibliography

1. Aird, W.C., *The role of the endothelium in severe sepsis and multiple organ dysfunction syndrome.* Blood, 2003. **101**(10): p. 3765-77.
2. Levi, M. and H. Ten Cate, *Disseminated intravascular coagulation.* N Engl J Med, 1999. **341**(8): p. 586-92.
3. Qi, J., S. Goralnick, and D.L. Kreutzer, *Fibrin regulation of interleukin-8 gene expression in human vascular endothelial cells.* Blood, 1997. **90**(9): p. 3595-602.
4. Day, S.M., et al., *Macrovascular thrombosis is driven by tissue factor derived primarily from the blood vessel wall.* Blood, 2005. **105**(1): p. 192-8.
5. Dentali, F., E. Romualdi, and W. Ageno, *The metabolic syndrome and the risk of thrombosis.* Haematologica, 2007. **92**(3): p. 297-9.

6. Mertens, I. and L.F. Van Gaal, *Obesity, haemostasis and the fibrinolytic system.* Obes Rev, 2002. **3**(2): p. 85-101.
7. Lumeng, C.N., *Adipose tissue macrophages: a piece of the PAI of metabolic syndrome.* Sci Transl Med, 2010. **2**(20): p. 20ps7.
8. Yan, S.B., et al., *Low levels of protein C are associated with poor outcome in severe sepsis.* Chest, 2001. **120**(3): p. 915-22.
9. Bernard, G.R., et al., *Efficacy and safety of recombinant human activated protein C for severe sepsis.* N Engl J Med, 2001. **344**(10): p. 699-709.
10. Van den Berghe, G., et al., *Intensive insulin therapy in mixed medical/surgical intensive care units: benefit versus harm.* Diabetes, 2006. **55**(11): p. 3151-9.
11. Merz, T.M. and S. Finfer, *Pro/con debate: Is intensive insulin therapy targeting tight blood glucose control of benefit in critically ill patients?* Crit Care, 2008. **12**(2): p. 212.
12. Turina, M., D.E. Fry, and H.C. Polk, Jr., *Acute hyperglycemia and the innate immune system: clinical, cellular, and molecular aspects.* Crit Care Med, 2005. **33**(7): p. 1624-33.
13. Otto, N.M., et al., *Hyperosmotic stress enhances cytokine production and decreases phagocytosis in vitro.* Crit Care, 2008. **12**(4): p. R107.
14. Bopp, C., et al., *Bench-to-bedside review: The inflammation-perpetuating pattern-recognition receptor RAGE as a therapeutic target in sepsis.* Crit Care, 2008. **12**(1): p. 201.
15. Goldin, A., et al., *Advanced glycation end products: sparking the development of diabetic vascular injury.* Circulation, 2006. **114**(6): p. 597-605.
16. Basta, G., A.M. Schmidt, and R. De Caterina, *Advanced glycation end products and vascular inflammation: implications for accelerated atherosclerosis in diabetes.* Cardiovasc Res, 2004. **63**(4): p. 582-92.
17. Bierhaus, A., et al., *Advanced glycation end product (AGE)-mediated induction of tissue factor in cultured endothelial cells is dependent on RAGE.* Circulation, 1997. **96**(7): p. 2262-71.
18. Boonen, E., et al., *Reduced cortisol metabolism during critical illness.* N Engl J Med, 2013. **368**(16): p. 1477-88.
19. Annane, D., et al., *A 3-level prognostic classification in septic shock based on cortisol levels and cortisol response to corticotropin.* JAMA, 2000. **283**(8): p. 1038-45.
20. Anagnostis, P., et al., *Clinical review: The pathogenetic role of cortisol in the metabolic syndrome: a hypothesis.* J Clin Endocrinol Metab, 2009. **94**(8): p. 2692-701.
21. Ridker, P.M., et al., *C-reactive protein levels and outcomes after statin therapy.* N Engl J Med, 2005. **352**(1): p. 20-8.
22. Dandona, P., A. Aljada, and A. Bandyopadhyay, *Inflammation: the link between insulin resistance, obesity and diabetes.* Trends Immunol, 2004. **25**(1): p. 4-7.
23. Meydan, C., S. Shenhar-Tsarfaty, and H. Soreq, *MicroRNA Regulators of Anxiety and Metabolic Disorders.* Trends Mol Med, 2016. **22**(9): p. 798-812.
24. Koch, A., et al., *Serum resistin levels in critically ill patients are associated with inflammation, organ dysfunction and metabolism and may predict survival of non-septic patients.* Crit Care, 2009. **13**(3): p. R95.
25. Sunden-Cullberg, J., et al., *Pronounced elevation of resistin correlates with severity of disease in severe sepsis and septic shock.* Crit Care Med, 2007. **35**(6): p. 1536-42.
26. Steppan, C.M., et al., *The hormone resistin links obesity to diabetes.* Nature, 2001. **409**(6818): p. 307-12.

27. Savage, D.B., et al., *Resistin / Fizz3 expression in relation to obesity and peroxisome proliferator-activated receptor-gamma action in humans.* Diabetes, 2001. **50**(10): p. 2199-202.
28. Stouthard, J.M., et al., *Interleukin-6 stimulates coagulation, not fibrinolysis, in humans.* Thromb Haemost, 1996. **76**(5): p. 738-42.
29. Bevilacqua, M.P., et al., *Recombinant tumor necrosis factor induces procoagulant activity in cultured human vascular endothelium: characterization and comparison with the actions of interleukin 1.* Proc Natl Acad Sci U S A, 1986. **83**(12): p. 4533-7.
30. Colotta, F., et al., *Expression of monocyte chemotactic protein-1 by monocytes and endothelial cells exposed to thrombin.* Am J Pathol, 1994. **144**(5): p. 975-85.
31. Vouret-Craviari, V., et al., *Regulation of the actin cytoskeleton by thrombin in human endothelial cells: role of Rho proteins in endothelial barrier function.* Mol Biol Cell, 1998. **9**(9): p. 2639-53.
32. Skurk, T. and H. Hauner, *Obesity and impaired fibrinolysis: role of adipose production of plasminogen activator inhibitor-1.* Int J Obes Relat Metab Disord, 2004. **28**(11): p. 1357-64.
33. Hess, K. and P.J. Grant, *Inflammation and thrombosis in diabetes.* Thromb Haemost, 2011. **105 Suppl 1**: p. S43-54.
34. Yarmolinsky, J., et al., *Plasminogen activator inhibitor-1 and type 2 diabetes: a systematic review and meta-analysis of observational studies.* Sci Rep, 2016. **6**: p. 17714.
35. Sola, E., et al., *Activated protein C levels in obesity and weight loss influence.* Thromb Res, 2009. **123**(5): p. 697-700.
36. Teruel-Montoya, R., F.R. Rosendaal, and C. Martinez, *MicroRNAs in hemostasis.* J Thromb Haemost, 2015. **13**(2): p. 170-81.
37. Sunderland, N., et al., *MicroRNA Biomarkers and Platelet Reactivity: The Clot Thickens.* Circ Res, 2017. **120**(2): p. 418-435.
38. Jickling, G.C., et al., *microRNA expression in peripheral blood cells following acute ischemic stroke and their predicted gene targets.* PLoS One, 2014. **9**(6): p. e99283.
39. Wang, H.J., et al., *Serum miR-122 levels are related to coagulation disorders in sepsis patients.* Clin Chem Lab Med, 2014. **52**(6): p. 927-33.
40. Investigators, N.-S.S., et al., *Intensive versus conventional glucose control in critically ill patients.* N Engl J Med, 2009. **360**(13): p. 1283-97.
41. Preiser, J.C., et al., *A prospective randomised multi-centre controlled trial on tight glucose control by intensive insulin therapy in adult intensive care units: the Glucontrol study.* Intensive Care Med, 2009. **35**(10): p. 1738-48.
42. Brunkhorst, F.M., et al., *Intensive insulin therapy and pentastarch resuscitation in severe sepsis.* N Engl J Med, 2008. **358**(2): p. 125-39.
43. Hillenbrand, A., et al., *Sepsis induced changes of adipokines and cytokines - septic patients compared to morbidly obese patients.* BMC Surg, 2010. **10**: p. 26.
44. Behnes, M., et al., *Alterations of adiponectin in the course of inflammation and severe sepsis.* Shock, 2012. **38**(3): p. 243-8.
45. Bornstein, S.R., et al., *Plasma leptin levels are increased in survivors of acute sepsis: associated loss of diurnal rhythm, in cortisol and leptin secretion.* J Clin Endocrinol Metab, 1998. **83**(1): p. 280-3.
46. Kantharidis, P., et al., *Diabetes complications: the microRNA perspective.* Diabetes, 2011. **60**(7): p. 1832-7.
47. Hamrahian, A.H., T.S. Oseni, and B.M. Arafah, *Measurements of serum free cortisol in critically ill patients.* N Engl J Med, 2004. **350**(16): p. 1629-38.

48. Ho, J.T., et al., *Septic shock and sepsis: a comparison of total and free plasma cortisol levels.* J Clin Endocrinol Metab, 2006. **91**(1): p. 105-14.
49. Annane, D., et al., *Effect of treatment with low doses of hydrocortisone and fludrocortisone on mortality in patients with septic shock.* JAMA, 2002. **288**(7): p. 862-71.
50. Sprung, C.L., et al., *Hydrocortisone therapy for patients with septic shock.* N Engl J Med, 2008. **358**(2): p. 111-24.
51. Keh, D., et al., *Effect of Hydrocortisone on Development of Shock Among Patients With Severe Sepsis: The HYPRESS Randomized Clinical Trial.* JAMA, 2016. **316**(17): p. 1775-1785.
52. Duclos, M., et al., *Increased cortisol bioavailability, abdominal obesity, and the metabolic syndrome in obese women.* Obes Res, 2005. **13**(7): p. 1157-66.
53. Yu, C.Y., et al., *Genome-wide analysis of glucocorticoid receptor binding regions in adipocytes reveal gene network involved in triglyceride homeostasis.* PLoS One, 2010. **5**(12): p. e15188.
54. Goedecke, J.H., et al., *Glucocorticoid receptor gene expression in adipose tissue and associated metabolic risk in black and white South African women.* Int J Obes (Lond), 2015. **39**(2): p. 303-11.
55. Constantinopoulos, P., et al., *Cortisol in tissue and systemic level as a contributing factor to the development of metabolic syndrome in severely obese patients.* Eur J Endocrinol, 2015. **172**(1): p. 69-78.
56. Patrassi, G.M., et al., *Further studies on the hypercoagulable state of patients with Cushing's syndrome.* Thromb Haemost, 1985. **54**(2): p. 518-20.
57. Van Zaane, B., et al., *Hypercoagulable state in Cushing's syndrome: a systematic review.* J Clin Endocrinol Metab, 2009. **94**(8): p. 2743-50.
58. Robertson, S., et al., *MicroRNA-24 is a novel regulator of aldosterone and cortisol production in the human adrenal cortex.* Hypertension, 2013. **62**(3): p. 572-8.
59. de Guia, R.M., et al., *microRNA-379 couples glucocorticoid hormones to dysfunctional lipid homeostasis.* EMBO J, 2015. **34**(3): p. 344-60.
60. Xu, B., et al., *Dax-1 and steroid receptor RNA activator (SRA) function as transcriptional coactivators for steroidogenic factor 1 in steroidogenesis.* Mol Cell Biol, 2009. **29**(7): p. 1719-34.
61. Esau, C., et al., *miR-122 regulation of lipid metabolism revealed by in vivo antisense targeting.* Cell Metab, 2006. **3**(2): p. 87-98.
62. Zampetaki, A. and M. Mayr, *MicroRNAs in vascular and metabolic disease.* Circ Res, 2012. **110**(3): p. 508-22.
63. Elmen, J., et al., *LNA-mediated microRNA silencing in non-human primates.* Nature, 2008. **452**(7189): p. 896-9.
64. Ward, J., et al., *Circulating microRNA profiles in human patients with acetaminophen hepatotoxicity or ischemic hepatitis.* Proc Natl Acad Sci U S A, 2014. **111**(33): p. 12169-74.
65. Janssen, H.L., et al., *Treatment of HCV infection by targeting microRNA.* N Engl J Med, 2013. **368**(18): p. 1685-94.
66. Luna, J.M., et al., *Hepatitis C virus RNA functionally sequesters miR-122.* Cell, 2015. **160**(6): p. 1099-110.
67. Correia, C.N., et al., *Circulating microRNAs as Potential Biomarkers of Infectious Disease.* Front Immunol, 2017. **8**: p. 118.

68. Caserta, S., et al., *Circulating Plasma microRNAs can differentiate Human Sepsis and Systemic Inflammatory Response Syndrome (SIRS).* Sci Rep, 2016. **6**: p. 28006.
69. Xiao, C., et al., *MiR-150 controls B cell differentiation by targeting the transcription factor c-Myb.* Cell, 2007. **131**(1): p. 146-59.
70. Ma, Y., et al., *Genome-wide sequencing of cellular microRNAs identifies a combinatorial expression signature diagnostic of sepsis.* PLoS One, 2013. **8**(10): p. e75918.
71. Vasilescu, C., et al., *MicroRNA fingerprints identify miR-150 as a plasma prognostic marker in patients with sepsis.* PLoS One, 2009. **4**(10): p. e7405.
72. Chou, C.F., et al., *KSRP ablation enhances brown fat gene program in white adipose tissue through reduced miR-150 expression.* Diabetes, 2014. **63**(9): p. 2949-61.
73. Ying, W., et al., *miR-150 regulates obesity-associated insulin resistance by controlling B cell functions.* Sci Rep, 2016. **6**: p. 20176.
74. Filios, S.R. and A. Shalev, *beta-Cell MicroRNAs: Small but Powerful.* Diabetes, 2015. **64**(11): p. 3631-44.
75. Melkman-Zehavi, T., et al., *miRNAs control insulin content in pancreatic beta-cells via downregulation of transcriptional repressors.* EMBO J, 2011. **30**(5): p. 835-45.
76. Zhang, D., et al., *miR-182 Regulates Metabolic Homeostasis by Modulating Glucose Utilization in Muscle.* Cell Rep, 2016. **16**(3): p. 757-68.
77. Poy, M.N., et al., *A pancreatic islet-specific microRNA regulates insulin secretion.* Nature, 2004. **432**(7014): p. 226-30.
78. Arner, P. and A. Kulyte, *MicroRNA regulatory networks in human adipose tissue and obesity.* Nat Rev Endocrinol, 2015. **11**(5): p. 276-88.
79. Karolina, D.S., et al., *Circulating miRNA profiles in patients with metabolic syndrome.* J Clin Endocrinol Metab, 2012. **97**(12): p. E2271-6.
80. Tang, W.F., et al., *Host MicroRNA miR-197 Plays a Negative Regulatory Role in the Enterovirus 71 Infectious Cycle by Targeting the RAN Protein.* J Virol, 2015. **90**(3): p. 1424-38.
81. Li, L.M., et al., *Serum microRNA profiles serve as novel biomarkers for HBV infection and diagnosis of HBV-positive hepatocarcinoma.* Cancer Res, 2010. **70**(23): p. 9798-807.
82. Rodriguez, A., et al., *Requirement of bic/microRNA-155 for normal immune function.* Science, 2007. **316**(5824): p. 608-11.
83. Nazari-Jahantigh, M., et al., *MicroRNA-155 promotes atherosclerosis by repressing Bcl6 in macrophages.* J Clin Invest, 2012. **122**(11): p. 4190-202.
84. Lin, X., et al., *MiR-155 Enhances Insulin Sensitivity by Coordinated Regulation of Multiple Genes in Mice.* PLoS Genet, 2016. **12**(10): p. e1006308.
85. Shaked, I., et al., *MicroRNA-132 potentiates cholinergic anti-inflammatory signaling by targeting acetylcholinesterase.* Immunity, 2009. **31**(6): p. 965-73.
86. Hanin, G., et al., *miRNA-132 induces hepatic steatosis and hyperlipidaemia by synergistic multitarget suppression.* Gut, 2017.
87. Geisler, S. and J. Coller, *RNA in unexpected places: long non-coding RNA functions in diverse cellular contexts.* Nat Rev Mol Cell Biol, 2013. **14**(11): p. 699-712.
88. Dey, B.K., A.C. Mueller, and A. Dutta, *Long non-coding RNAs as emerging regulators of differentiation, development, and disease.* Transcription, 2014. **5**(4): p. e944014.
89. Viereck, J., et al., *Long noncoding RNA Chast promotes cardiac remodeling.* Sci Transl Med, 2016. **8**(326): p. 326ra22.
90. Zur Bruegge, J., R. Einspanier, and S. Sharbati, *A Long Journey Ahead: Long Non-coding RNAs in Bacterial Infections.* Front Cell Infect Microbiol, 2017. **7**: p. 95.

91. Chen, Z., *Progress and prospects of long noncoding RNAs in lipid homeostasis.* Mol Metab, 2016. **5**(3): p. 164-70.
92. Gernapudi, R., et al., *MicroRNA 140 Promotes Expression of Long Noncoding RNA NEAT1 in Adipogenesis.* Mol Cell Biol, 2016. **36**(1): p. 30-8.
93. Wang, Z., et al., *NEAT1 modulates herpes simplex virus-1 replication by regulating viral gene transcription.* Cell Mol Life Sci, 2017. **74**(6): p. 1117-1131.
94. Ma, H., et al., *The Long Noncoding RNA NEAT1 Exerts Antihantaviral Effects by Acting as Positive Feedback for RIG-I Signaling.* J Virol, 2017. **91**(9).
95. Imamura, K., et al., *Long noncoding RNA NEAT1-dependent SFPQ relocation from promoter region to paraspeckle mediates IL8 expression upon immune stimuli.* Mol Cell, 2014. **53**(3): p. 393-406.
96. Khalil, A.M., et al., *Many human large intergenic noncoding RNAs associate with chromatin-modifying complexes and affect gene expression.* Proc Natl Acad Sci U S A, 2009. **106**(28): p. 11667-72.
97. He, S., et al., *MicroRNA-encoding long non-coding RNAs.* BMC Genomics, 2008. **9**: p. 236.
98. Gong, J., et al., *lncRNASNP: a database of SNPs in lncRNAs and their potential functions in human and mouse.* Nucleic Acids Res, 2015. **43**(Database issue): p. D181-6.
99. Hanin, G., et al., *Competing targets of microRNA-608 affect anxiety and hypertension.* Hum Mol Genet, 2014. **23**(17): p. 4569-80.
100. Zhang, A.Q., et al., *Genetic variants of microRNA sequences and susceptibility to sepsis in patients with major blunt trauma.* Ann Surg, 2015. **261**(1): p. 189-96.
101. Wu, H., et al., *LncRNA-HOTAIR promotes TNF-alpha production in cardiomyocytes of LPS-induced sepsis mice by activating NF-kappaB pathway.* Biochem Biophys Res Commun, 2016. **471**(1): p. 240-6.
102. Zgheib, C., et al., *Long non-coding RNA Lethe regulates hyperglycemia-induced reactive oxygen species production in macrophages.* PLoS One, 2017. **12**(5): p. e0177453.
103. Rapicavoli, N.A., et al., *A mammalian pseudogene lncRNA at the interface of inflammation and anti-inflammatory therapeutics.* Elife, 2013. **2**: p. e00762.
104. Liu, Y., et al., *Tissue-specific RNA-Seq in human evoked inflammation identifies blood and adipose LincRNA signatures of cardiometabolic diseases.* Arterioscler Thromb Vasc Biol, 2014. **34**(4): p. 902-12.
105. Carpenter, S., et al., *A long noncoding RNA mediates both activation and repression of immune response genes.* Science, 2013. **341**(6147): p. 789-92.
106. Krawczyk, M. and B.M. Emerson, *p50-associated COX-2 extragenic RNA (PACER) activates COX-2 gene expression by occluding repressive NF-kappaB complexes.* Elife, 2014. **3**: p. e01776.
107. Wang, P., et al., *The STAT3-binding long noncoding RNA lnc-DC controls human dendritic cell differentiation.* Science, 2014. **344**(6181): p. 310-3.
108. Li, Z., et al., *The long noncoding RNA THRIL regulates TNFalpha expression through its interaction with hnRNPL.* Proc Natl Acad Sci U S A, 2014. **111**(3): p. 1002-7.
109. Vereecke, L., R. Beyaert, and G. van Loo, *The ubiquitin-editing enzyme A20 (TNFAIP3) is a central regulator of immunopathology.* Trends Immunol, 2009. **30**(8): p. 383-91.
110. Karpe, F. and K.E. Pinnick, *Biology of upper-body and lower-body adipose tissue--link to whole-body phenotypes.* Nat Rev Endocrinol, 2015. **11**(2): p. 90-100.
111. Rinn, J.L., et al., *Functional demarcation of active and silent chromatin domains in human HOX loci by noncoding RNAs.* Cell, 2007. **129**(7): p. 1311-23.
112. Tsai, M.C., et al., *Long noncoding RNA as modular scaffold of histone modification complexes.* Science, 2010. **329**(5992): p. 689-93.

113. Korostowski, L., N. Sedlak, and N. Engel, *The Kcnq1ot1 long non-coding RNA affects chromatin conformation and expression of Kcnq1, but does not regulate its imprinting in the developing heart.* PLoS Genet, 2012. **8**(9): p. e1002956.
114. Moran, I., et al., *Human beta cell transcriptome analysis uncovers lncRNAs that are tissue-specific, dynamically regulated, and abnormally expressed in type 2 diabetes.* Cell Metab, 2012. **16**(4): p. 435-48.
115. Vausort, M., D.R. Wagner, and Y. Devaux, *Long noncoding RNAs in patients with acute myocardial infarction.* Circ Res, 2014. **115**(7): p. 668-77.
116. Sun, J., et al., *Differentially expressed circulating LncRNAs and mRNA identified by microarray analysis in obese patients.* Sci Rep, 2016. **6**: p. 35421.
117. Alvarez-Dominguez, J.R., et al., *De Novo Reconstruction of Adipose Tissue Transcriptomes Reveals Long Non-coding RNA Regulators of Brown Adipocyte Development.* Cell Metab, 2015. **21**(5): p. 764-76.
118. Zhao, X.Y. and J.D. Lin, *Long Noncoding RNAs: A New Regulatory Code in Metabolic Control.* Trends Biochem Sci, 2015. **40**(10): p. 586-96.
119. Kumarswamy, R., et al., *Circulating long noncoding RNA, LIPCAR, predicts survival in patients with heart failure.* Circ Res, 2014. **114**(10): p. 1569-75.
120. Kallen, A.N., et al., *The imprinted H19 lncRNA antagonizes let-7 microRNAs.* Mol Cell, 2013. **52**(1): p. 101-12.
121. Hube, F., et al., *Steroid receptor RNA activator protein binds to and counteracts SRA RNA-mediated activation of MyoD and muscle differentiation.* Nucleic Acids Res, 2011. **39**(2): p. 513-25.
122. Yan, B., et al., *lncRNA-MIAT regulates microvascular dysfunction by functioning as a competing endogenous RNA.* Circ Res, 2015. **116**(7): p. 1143-56.
